# Case Report: A case of Fournier’s gangrene of the scrotum

**DOI:** 10.3389/fmed.2026.1791234

**Published:** 2026-04-08

**Authors:** Ling Yuan, Yingying Sun, Jianjun Yan

**Affiliations:** Department of Dermatology, Shandong Provincial Hospital Affiliated to Shandong First Medical University, Jinan, Shandong, China

**Keywords:** Fournier’s gangrene, necrotizing fasciitis, soft tissue infection, vacuum sealing drainage, wound managemen

## Abstract

A 68-year-old man with poorly controlled type 2 diabetes and untreated stage 2 hypertension presented with a 7-day history of painful scrotal and perianal swelling. Imaging revealed subcutaneous gas and necrotic sinus tracts, and tissue cultures showed a polymicrobial infection. Fournier’s gangrene was diagnosed. Prompt surgical debridement, vacuum sealing drainage (VSD), glycemic control, and antibiotic therapy led to complete recovery, highlighting the importance of early recognition and aggressive management.

## Highlights

- Fournier’s gangrene is a rapidly progressive necrotizing soft tissue infection, with poorly controlled diabetes being a major predisposing factor.
- This case uniquely illustrates the complementary roles of ultrasound, CT, and MRI in accurately identifying subcutaneous gas and necrotic sinus tract extension.
- A polymicrobial infection confirmed the need for early broad-spectrum antimicrobial therapy.
- Prompt surgical debridement combined with vacuum sealing drainage and strict glycemic control led to successful wound healing, emphasizing the value of a multidisciplinary approach.

## Clinical history

A 68-year-old man presented to the emergency department with a 7-day history of painful swelling in the scrotum and perianal region. He had a more than 10-year history of type 2 diabetes, with irregular use of hypoglycemic drugs and poor glycemic control. He also had a 10-year history of stage 2 hypertension that had remained untreated.

### Physical examination

**Figure 1:**
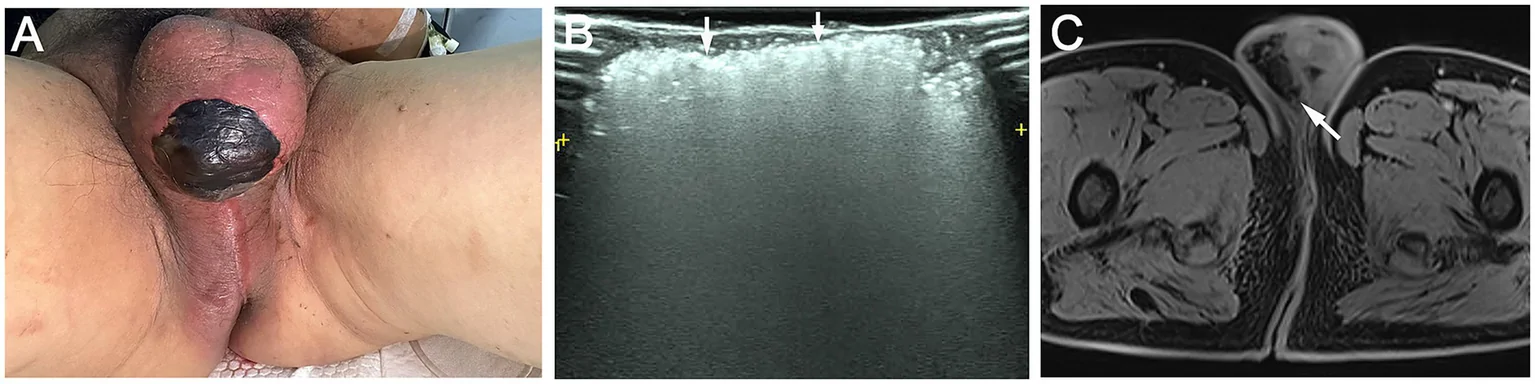
Skin lesions of the scrotum and perianal area **(A)**; air in the subcutaneous tissue of the scrotum and perianal region **(B)**; a sinus tract formed by subcutaneous necrosis extending from the scrotum to the perianal area (**C**, arrow).

Physical examination revealed swelling and erythema of the scrotum and perianal area, with a black scab covering the surface of the scrotum, without skin ulceration, erosion, exudate, or suppuration (Figure 1A).

### Imaging and microbiological findings

Ultrasound examination revealed air in the subcutaneous tissue of the scrotum and perianal region, which was further confirmed by magnetic resonance imaging (MRI), and found a sinus tract formed by subcutaneous necrosis extending from the scrotum to the perianal area (Figures 1B,C, arrow). Bacterial and fungal cultures of necrotic tissue showed mixed growth of *Enterococcus*, *Klebsiella pneumoniae*, and *Corynebacterium* (Supplementary Figure 1). We also performed antimicrobial susceptibility testing on the cultured bacteria from the patient, and the minimum inhibitory concentration (MIC) results are presented in Table 1.

**Table 1:** MIC results for *Enterococcus*.

Antibiotic name	Method	Value	Interpretation	Sensitivity (S)	Resistance (R)
Macrolides					
Erythromycin	MIC	> = 8	Resistant (R)	≤0.5	≥8
Chainsomycin					
Quinupristin	MIC	4	Resistant (R)	≤1	≥4
Penicillins					
Penicillin G	MIC	0.25	Sensitive (S)	≤8	≥16
Ampicillin	MIC	<=2	Sensitive (S)	≤8	≥16
Amoxicillin	*		Sensitive (S)		
Tetracycline					
Tigecycline	MIC	<=0.12	Sensitive (S)	≤0.25	
Glycopeptides					
Vancomycin	MIC	<=0.5	Sensitive (S)	≤4	≥32
Oxazolidinones					
Linezolid	MIC	2	Sensitive (S)	≤2	≥8

MIC, minimum inhibitory concentration.

Antimicrobial Susceptibility Testing Results: *Enterococcus avium*.

*Susceptibility inferred from ampicillin results.

## What is your diagnosis?

Fournier’s gangrene is a specific type of necrotizing fasciitis, associated with infection by *Enterococcus*, *Klebsiella pneumoniae*, and *Corynebacterium*.

## Treatment process

Upon admission, the patient was immediately evaluated using the Fournier’s Gangrene Severity Index (FGSI), with an initial score of 4. Given the rapid clinical progression, empirical broad-spectrum antibiotic therapy with meropenem (1 g every 8 h) and vancomycin (1 g every 12 h) was promptly initiated. In view of the patient’s poorly controlled diabetes, intensive insulin therapy was administered to achieve optimal glycemic control.

Considering the severity of the condition, emergency surgical debridement was performed. Intraoperatively, an undermined cavity was identified at the 12 o’clock position relative to the anal verge. After meticulous and extensive debridement of all necrotic tissue Supplementary Figure 2, vacuum sealing drainage (VSD) was applied, with continuous negative pressure maintained between 0.02 and 0.04 MPa. Intraoperative specimens were obtained for bacterial culture and antimicrobial susceptibility testing. The patient had a 10-year history of hypertension without any prior pharmacological treatment. Following the initial debridement procedure, the patient exhibited a reactive elevation in blood pressure, with readings ranging from 188 to 200 mmHg and 115 to 120 mmHg. After consulting with the cardiology department, benidipine was initiated to manage blood pressure control.

Based on microbiological findings and the postoperative clinical course, the antibiotic regimen was subsequently adjusted to ceftizoxime (3 g, twice daily, intravenous infusion), and batroxobin was administered for hemostatic support. The patient underwent a total of four sequential surgical debridement procedures, each followed by VSD therapy. During the fifth procedure, as the wound demonstrated satisfactory granulation and progressive healing, only trimming of hyperplastic granulation tissue was performed, and VSD was not reapplied.

The postoperative course was uneventful. The patient remained hemodynamically stable and afebrile. After three consecutive days of routine wound dressing changes, he was discharged with instructions for continued outpatient wound care and follow-up.

## Discussion

In 1883, Fournier described a clinical entity characterized by the sudden onset of painful scrotal swelling, which rapidly progressed to gangrene. Fournier’s gangrene is a life-threatening, rapidly progressive necrotizing soft tissue infection (NSTI) of the external genitalia and/or perineum (1). This is a distinct type of necrotizing fasciitis, characterized by rapid tissue destruction caused by the synergistic action of bacteria and the symbiosis of multiple microorganisms (2). Diabetes, trauma, liver and kidney diseases, immunosuppressive drugs, and human immunodeficiency virus infection are recognized risk factors (3). In the differential diagnosis, gas gangrene should be carefully distinguished from Fournier’s gangrene. Gas gangrene is primarily characterized by rapidly progressive myonecrosis with a fulminant clinical course, often worsening within hours. It typically presents with extensive gas production, leading to prominent crepitus and severe systemic toxicity (4). In contrast, although subcutaneous gas and crepitus may also occur in Fournier’s gangrene, muscle involvement is usually limited, and the infection predominantly affects the fascia. Therefore, the presence of extensive muscle necrosis with disproportionate systemic toxicity favors a diagnosis of gas gangrene rather than Fournier’s gangrene. The literature describes similar clinical scenarios where patients presented with polymicrobial infections, including *Morganella* and *Klebsiella* (5), alongside fungal involvement by organisms such as *Actinomyces* (6, 7).

Physical examination findings (fever, hemorrhagic bullae, necrosis, scabbing, and hypotension), FGSI scores, imaging results, and microbiological analyses are all valuable for the early identification of the disease (8).

After debridement and removal of necrotic tissue, combined with VSD, glycemic control, fluid resuscitation, and antimicrobial therapy, the wound healed, and the patient was discharged. Extensive evidence suggests that the synergy of integrated antimicrobial protocols, vacuum-assisted closure, and tailored antibiotic therapy optimizes clinical outcomes and significantly reduces mortality (9).

This case highlights the importance of early diagnosis and treatment in reducing disease-related mortality. Rapid resuscitation, prompt antibiotic therapy, and early, aggressive surgical debridement are the key elements of initial management.

## Data Availability

The original contributions presented in the study are included in the article/Supplementary material, further inquiries can be directed to the corresponding author.
