# Correction: Case Report: A case of Fournier's gangrene of the scrotum

**DOI:** 10.3389/fmed.2026.1986579

**Published:** 2026-09-15

**Authors:** Ling Yuan, Yingying Sun, Jianjun Yan

**Affiliations:** Department of Dermatology, Shandong Provincial Hospital Affiliated to Shandong First Medical University, Jinan, Shandong, China

**Keywords:** fournier's gangrene, necrotizing fasciitis, soft tissue infection, vacuum sealing drainage, wound management

The funder Shandong Provincial Natural Science Foundation was erroneously omitted. The corrected Funding statement is:

“The author(s) declared that financial support was received for this work and/or its publication. This work was supported by the Shandong Provincial Natural Science Foundation (No. ZR2025MS1457).”

The original version of this article has been updated.

