# Supplementary material for: Case Report: A case of Fournier’s gangrene of the scrotum
Source: Front Med (Lausanne). 2026 Apr 8;13:1791234. doi: 10.3389/fmed.2026.1791234 (PMC13099282; doi:10.3389/fmed.2026.1791234)
Supplement: Supplementary file 1 [file Supplementary_file_1.docx]

# Supplemental Materials


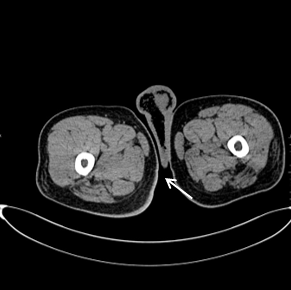


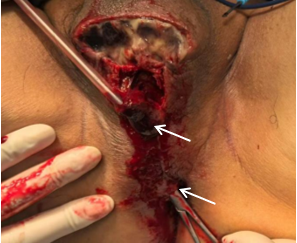
Supplementary figure 1: Pelvic computed tomography (CT). CT examination revealed air in the subcutaneous of scrotum and perianal region (arrow), consistent with skin and soft tissue infection of scrotum and perianal.

Supplementary figure 2: Surgical removal of necrotic tissue thoroughly. Necrotic sinus tract was found in the subcutaneous tissue from scrotum to perianal area during surgery (arrow), which was consistent with the result of magnetic resonance imaging examination.
